# Case Report: Bionic Reconstruction in an Adult With Obstetric Brachial Plexus Injury

**DOI:** 10.3389/fresc.2021.804376

**Published:** 2022-01-05

**Authors:** Anna Boesendorfer, Agnes Sturma, Clemens Gstoettner, Anna Pittermann, Gregor Laengle, Oskar C. Aszmann

**Affiliations:** ^1^Clinical Laboratory for Bionic Extremity Reconstruction, Department of Plastic, Reconstructive and Aesthetic Surgery, Medical University of Vienna, Vienna, Austria; ^2^Neurorehabilitation Engineering Group, Department of Bioengineering, Imperial College London, London, United Kingdom; ^3^Department of Plastic, Reconstructive and Aesthetic Surgery, Medical University of Vienna, Vienna, Austria; ^4^Department of Clinical Psychology, General Hospital of Vienna, Vienna, Austria

**Keywords:** obstetric brachial plexus injury (OBPI), bionic reconstruction, upper limb amputation, prosthesis, case report, functional outcome

## Abstract

**Introduction:** Many adults who had a severe Narakas IV obstetric brachial plexus injury (OBPI) suffer from extensive impairments in daily living due to limited hand-arm function. The dramatic loss of axonal support at this very early age of development often render the entire extremity a biologic wasteland and reconstructive methods and therapies often fail to recover any functional hand use. In this scenario bionic reconstruction, including an elective amputation and a subsequent prosthetic fitting, may enable functional improvement in adults suffering from the consequences of such severe brachial plexus injuries. We here describe our experience in treating such patients and lay out the surgical rational and rehabilitation protocol exemplified in one patient.

**Case Presentation/Methods:** A 27-year-old adult with a unilateral OBPI contacted our center. He presented with globally diminished function of the affected upper extremity with minimal hand activity, resulting in an inability to perform various tasks of daily living. No biological reconstructive efforts were available to restore meaningful hand function. An interdisciplinary evaluation, including a psychosocial assessment, was used to assess eligibility for bionic reconstruction. Before the amputation and after the prosthetic fitting functional assessments and self-reported questionnaires were performed.

**Results:** One month after the amputation and de-rotation osteotomy of the humerus the patient was fitted with a myoelectric prosthesis. At the 1.5 year-follow-up assessment, the patient presented with a distinct improvement of function: the ARAT improved from 12 to 20 points, SHAP score improved from 8 to 29, and the DASH value improved from 50 to 11.7. The average wearing times of the prosthesis were 5 to 6 h per day (on 4–5 days a week).

**Discussion:** The options for adults suffering from the consequences of severe OBPIs to improve function are limited. In selected patients in whom the neurological deficit is so severe that biologic hand function is unsatisfactory, an elective amputation and subsequent restoration of the hand with mechatronic means may be an option. The follow-up results indicate that this concept can indeed lead to solid hand function and independence in daily activities after amputation, subsequent prosthetic fitting, and rehabilitation.

## Introduction

Obstetric brachial plexus injuries (OBPI) refer to injuries of the brachial plexus that occur during delivery (1). The incidence of OBPI is documented in Norway with 0.3%, with a relatively high recovery rate, nonetheless one in every 2,000 babies has to live with a permanent injury of the plexus (2). Guidelines for patients with OBPI recommend early referral to multidisciplinary centers (at 1 month of age) (3). If no recovery occurs, early surgery is indicated at 3–9 months after birth, depending on the extent and severity of the injury (4).

If these early interventions do to not lead to sufficient outcomes, only a few surgical interventions are available after adolescence. They include the modified Quad surgery (5), tendon transfers for restoration of external shoulder rotation, and humeral rotational osteotomy in combination with lengthening (6). These limited options are reflected by the impaired hand function described by many adults after severe OBPI. Common clinical findings include problems in performing daily activities due to a lack of useful hand function, a high prevalence of pain as well as reduced sensation, arthritis, and an overall reduced quality of life (1, 7, 8). Despite their perceived disability, this patient group rarely receives rehabilitation measures (1) which might be related to the limited options available.

Recently, the method of “bionic reconstruction” has expanded options for patients with a very limited upper limb function. The procedure includes elective amputation of the hand, de-rotation osteotomy of the humerus for better positioning of the forearm, followed by prosthetic fitting. The feasibility of bionic reconstructions in patients suffering from brachial plexus injuries in adulthood is well-documented (9–11). However, studies investigating this treatment after OBPI are not found in literature. The aim of this report is to present a further indication for this procedure in patients who suffer the consequence of severe birth related plexus lesions. We report the case of a young patient with a unilateral OBPI who underwent bionic reconstruction, including long-term functional outcomes.

## Case Description

In July 2019, a 27-year-old adult with history of a right-sided Narakas IV OBPI contacted our center with the wish for bionic reconstruction. The patient described himself as male. In the first year after his birth, reconstruction of the brachial plexus was performed, including direct replantation of the lower roots C8 and T1 to the spine. An improvement of function was documented in his medical report. During childhood and adolescence, the patient did not receive any therapeutical interventions regarding his OBPI. He was unsatisfied with his situation when approaching our center and described his arm as an “annoying appendix being in his way.”

## Methods

**Figure 1:**
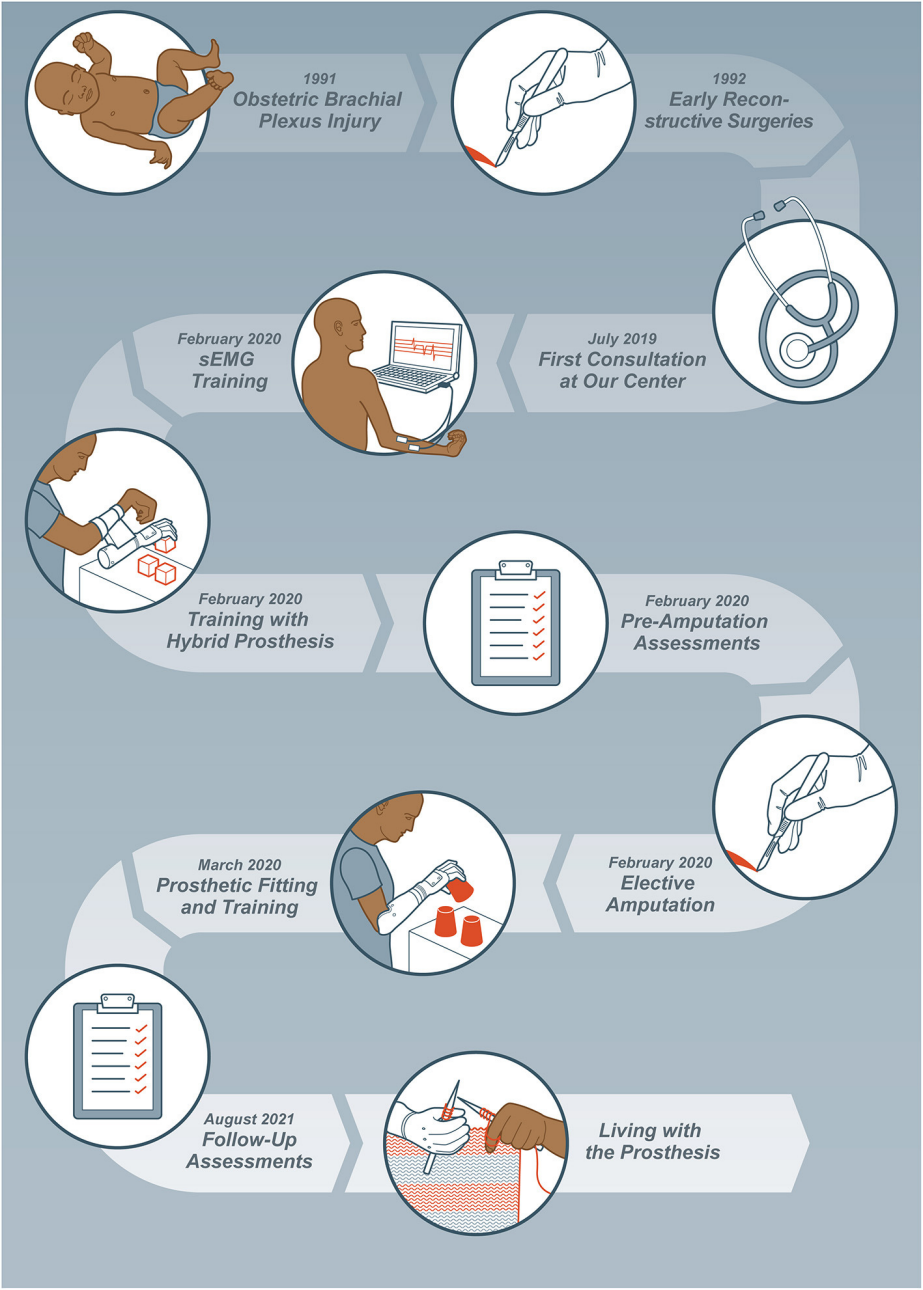
Timeline of the patient undergoing bionic reconstruction.

After the patient presented at our center, different possibilities were discussed, and it was decided that further biological reconstructive efforts would not lead to favorable outcomes and that bionic reconstruction should be evaluated. Therefore, the previously established guidelines for the procedure including a psychological assessment were followed (12, 13). Inclusion and exclusion criteria for bionic reconstruction have been described previously (11, 13). Furthermore, the patient received 6 days of intensive rehabilitation and home training by a physical and occupational therapist (AS and AB) with details outlined below and depicted in Figure 1. The patient gave written informed consent to take part in this study and standardized guidelines for reporting the case report (CARE checklist) were used (refer to Supplementary Material 1).

### Clinical Examination

**Figure 2:**
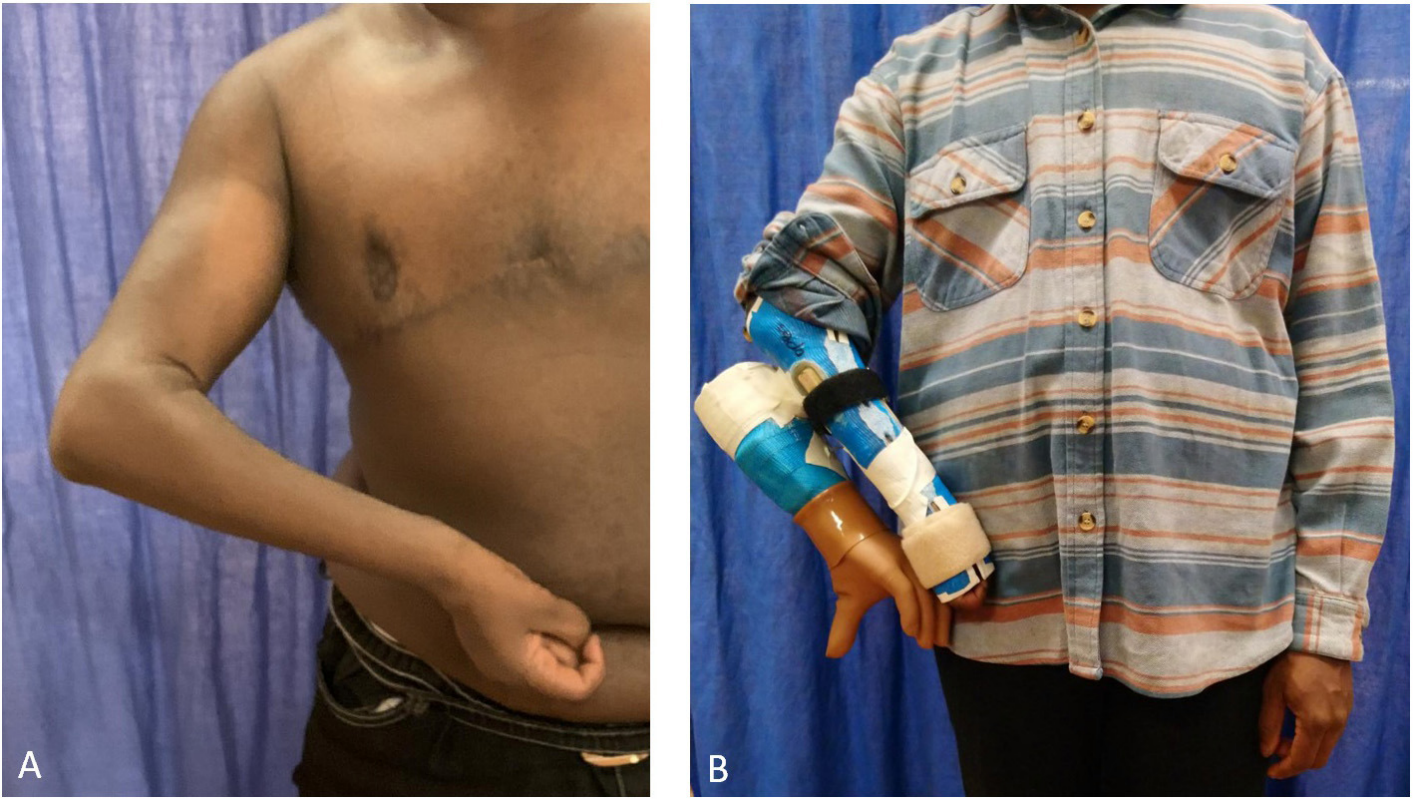
Plexus arm **(A)** and hybrid prosthesis that is mounted on the paretic arm **(B)**.

The right arm presented hypoplastic with an internal rotation deformity at shoulder level and flexion contracture in the elbow (see Figure 2A). The fingers and thumb were fixed in a flexed position, but minimal flexion of the thumb was possible. The patient was able to clamp small objects (for instance a wooden cube 2 × 2 × 2 cm) between his thumb and fingers, however, had issues releasing them. There was minimal active movement of the wrist in extension and flexion. Active shoulder abduction was 80° and flexion was 110°. Active elevation of the arm with evasion movement was possible to 150°. The elbow showed a passive extension deficit of 75° and active flexion of up to 100°. The patient presented without any useful sensation in the hand and forearm.

### Surface Electromyography Biofeedback Training and Training With a Table-Top Prosthesis

In a first step, surface electromyography (sEMG) signals on the forearm were identified [following established protocols (12, 14)] (by AS and AB). This was done by using an sEMG biofeedback system, where the muscle activation could be observed on a screen. Various electrode positions on the forearm and movement commands (like closing the fingers, flexing the wrist, opening the hand, extending single fingers, etc.) were tested. The aim was to find two different sEMG signals, one signal for opening the prosthesis and another for closing the hand. After the identification of the most appropriate electrode positions and movement cues (for the patient the best cues were flexing the fingers and extension of the thumb), these were trained separately. The patient was asked to activate one signal while the other remained relaxed and vice versa with a rest period in between. As soon as the activation of the signals could be reliably performed, the movements were practiced with a table-top prosthesis (opening and closing of the hand). This allowed the patient to receive direct feedback regarding movement intention and subsequent prosthetic motion.

### Fitting and Training With Hybrid Prosthesis

A hybrid prosthesis that could be attached on the paretic arm with the pre-defined electrode positions was initially fitted (see Figure 2B). Intensive training with the device (12, 14) started with opening and closing of the hand in various speeds and different positions (standing, sitting, different arm positions). In a second step, grasping and manipulation of objects was trained in therapy and at home. Finally, simple tasks of daily living could be trained with the hybrid prosthesis, as a proof-of-concept. This also allowed the patient to experience limitations of current prosthetic devices (such as the lack of sensory feedback) before final decision making. Additional effects of the training were strengthening of the biceps and shoulder muscles.

### Psychosocial Assessment

In a semi-structured interview the psychologist (AP) assessed the overall psychosocial status, the patient's motivation for the amputation, and the expectations of the outcome following the guidelines outlined by Hruby et al. (13). One of the major points thereby always addressed is the fact that an amputation is an irreversible procedure and that a prosthesis is only a tool which cannot be compared with an intact biological hand. As the patient was assessed as psychologically stable, not meeting any exclusion criteria and being aware of the consequences of the procedure, clearance for the planned amputation was given.

### Functional Assessment

The current status and function of the arm and hand were assessed with standardized assessments including the “Action Research Arm Test” (ARAT) and “Southampton Hand Assessment Procedure” (SHAP) by a physical and occupational therapist (by AS and AB). The ARAT assesses the function of the impaired upper limb in 19 tasks using grasping and manipulation of various objects, as well as gross motor movements. The highest score, indicating no impairment, is 57 and the lowest score, indicating no function, is 0 (15). The SHAP test is designed to assess prosthetic function, also including grasping and manipulation of objects and tasks of daily living (such as opening a jar, undoing buttons, etc.). The time for each task is measured and determines the overall functional score, with 100 indicating normal function and 0 indicating no function (16). Both tests were conducted in a standing position. First, the native function of the affected hand was tested, afterwards the same tests were conducted with the hybrid prosthesis attached. As these functional tests using the hybrid prosthesis indicated acceptable prosthetic control, amputation was considered suitable from a functional perspective as well. Additionally, the “Disabilities of the Arm, Shoulder and Hand” (DASH) questionnaire, which assesses the limitations in everyday life due to an injury of the upper extremity, was completed by the patient. Here, 0 corresponds to no disability and 100 shows a complete dependence in daily life (17, 18). The current pain level was documented as well by using the visual analog scale (VAS) (100 mm line). 1.5 years after the final prosthesis fitting these assessments were performed again and a semi-structured interview was conducted. Additionally, some open questions and rating questions on an 11-level numeric rating scale (NRS) (0 means disagree/never and 10 agree/always) regarding the use of the prosthesis and the prosthesis embodiment were asked (11).

### Surgery

After the approval from the multidisciplinary team the surgery took place in the same month. The procedure included a de-rotation osteotomy of the humerus, a shortening of the olecranon to release the extension deficit in the elbow and the transradial amputation (performed by OCA).

### Prosthetic Fitting and Prosthesis Training

**Figure 3:**
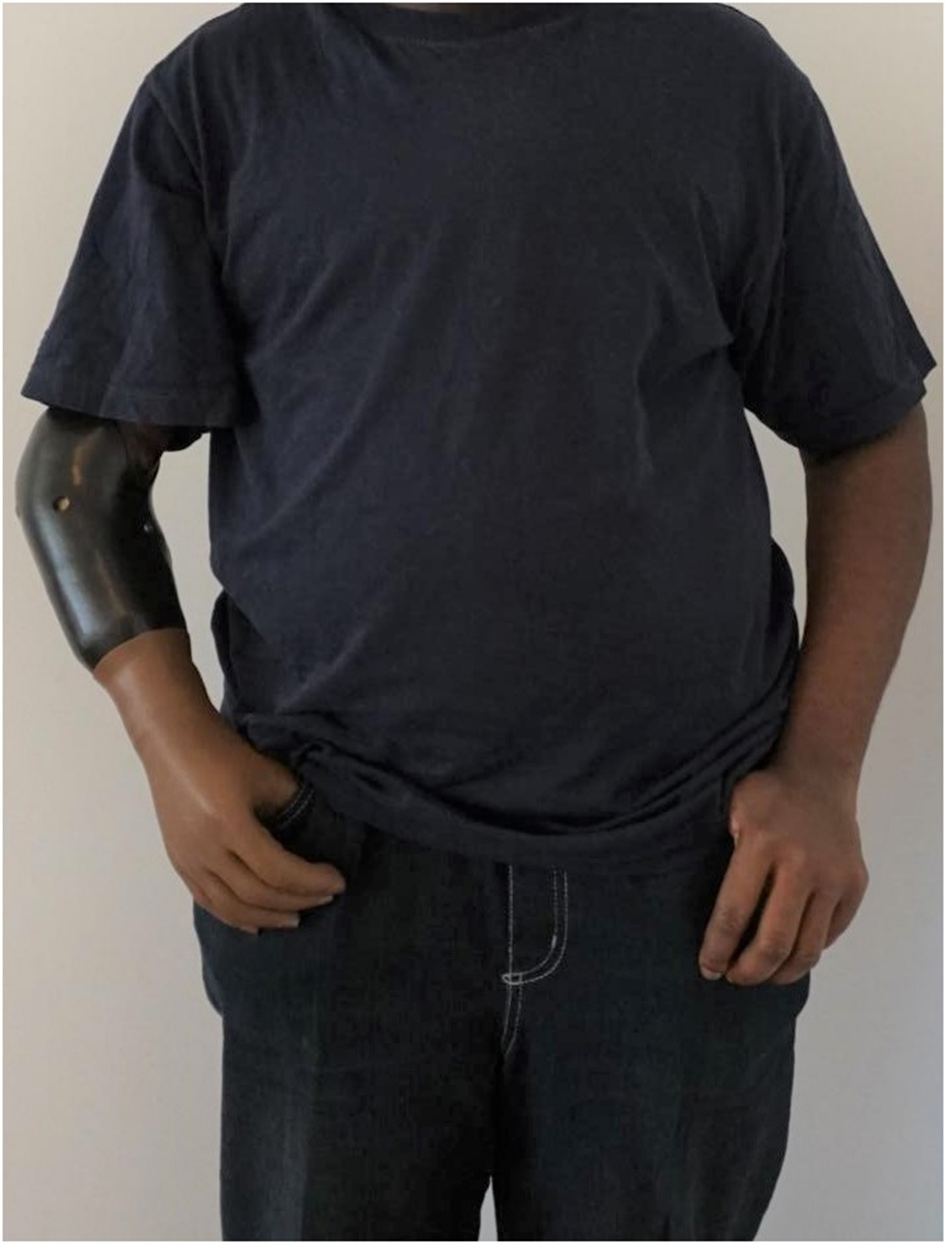
Patient with the final prosthetic fitting 1.5 years after the amputation.

The rehabilitation process was started by a rehabilitation physician in the home country of the patient in March 2020. The rehabilitation team consisted of occupational/physical therapists, prosthetist and physician. After the surgical wounds had healed the patient received a prosthetic fitting with a MyoHand VariPlus Speed (Ottobock, Duderstadt, Germany) (see Figure 3). He attended a weekly prosthetic training (30 min per session) by an occupational/physical therapist for ~1 year and trainings ongoing. The therapy consisted of simple movements of the prosthesis (opening/closing) in different speeds and positions, and further training of grasping and manipulation of different abstract objects. In a last step activities of daily living were trained with the prosthesis (including knitting). Also exercises for strengthening, endurance and symmetry of the body were discussed. The patient stayed in contact with our team *via* email and video calls.

## Results

As shown in Table 1, the ARAT improved from 12 pre-operatively to 20 at 1.5 years after the surgery, the SHAP test from 8 to 29 and the DASH showed an improvement from 50 to 11.7 (raw data can be found in the Supplementary Material 2, videos from one ARAT task can be found in the Supplementary Material 3). The testing with the hybrid prosthesis had already indicated an improvement of hand function compared to the biological arm with an ARAT score of 17 and a SHAP score of 19. The patient described no pain before and after the bionic reconstruction. The extension deficit of the elbow improved through treatment, with a final range of motion of 0°-55°-100°.

**Table 1:** Results of the assessments with the plexus hand, the hybrid prosthesis and the final prosthesis (1.5 years follow-up).

	**Plexus hand**	**Hybrid prosthesis**	**1.5 years follow-up with prosthesis**
DASH	50	-	11.7
ARAT	12	17	20
SHAP	8	19	29
Pain (VAS)	0	-	0
ROM elbow flexion	0°-75°-100°	-	0°-55°-100°

*A higher score for the ARAT and SHAP test shows a better function whereas a lower score in the DASH questionnaire shows less disabilities. 0 means no pain on the visual analog scale (VAS). Because of the flexion contracture the range of motion (ROM) of the elbow is presented as no extension possible (0°)—minimal flexed position to maximum flexed position*.

The average wearing time of the prosthesis was 5–6 h per day (on ~4–5 days per week) and the patient reported a particular preference of wearing the prosthesis when leaving his house. The patient further reported to sometimes wear his prothesis switched off, activating it only when needed. He liked wearing the prosthesis a lot (NRS “9/10”). “I did bimanual tasks with my intact arm/hand together with my prosthesis” was rated by the patient with “9/10” (NRS). He had the feeling that the prosthesis was a part of the body (NRS “10/10”) and that his prosthesis looked like a real part of the body (NRS “9/10”). Also the statement “I felt the prosthesis only as a tool, and not as a part of my body” was rated with “9/10” on the NRS. “I felt that I had full control over the prosthesis” was rated with “9/10” on the NRS (also see Supplementary Material 2). The satisfaction of the current function was rated with “5/10” on the NRS. In the personal interview at follow-up, he described that he sometimes struggled to control the prosthesis and that he wished for a different prosthesis model (a multi-articulating hand with different grasping types). Nevertheless, with the prosthesis he was able to do things that were not possible before, such as holding and fixing objects, carrying a bag and even knitting. He perceived his quality of life much higher than before and told us he would undergo the procedure again. If he had the choice, he might even opt for bionic reconstruction sooner. No adverse or unanticipated events occurred during the process.

## Discussion

The clinical prognosis after OBPI depends particularly on the severity and extent of the injury (as classified by Narakas), early surgical interventions if needed, and subsequent rehabilitation (3). While the majority of patients develop good upper extremity function (19), in some cases the motoneuron loss is of such extent that the entire neuromuscular system will undergo fatty-fibrous degeneration leading to multiple joint contractures and deformities rendering the extremity with severe impairments and reduced quality of life (1, 7, 8). This was the situation of a 27-year-old adult who approached us for consultation in 2019. He reported a great disability in daily life due to a Narakas IV OBPI, with resulting socioeconomic limitations such as inability to complete nursing school. The efforts that had already been pursued to improve the situation did not lead to satisfactory results for the patient. Furthermore, surgical procedures to improve function in severe Narakas IV lesions of the plexus are limited and restoring meaningful hand function is challenging (4). A case report of three female adults undergoing a modified Quad surgery, which is a combination of muscle transpositions, resulted in an improvement of the total modified Mallet Score in two of them after the surgery (5). Another case report could demonstrate an improvement of shoulder function after an external rotation osteotomy and lengthening of the humerus documented with the modified Mallet Score (6). In both studies the impact of the intervention on hand and arm function in daily life activities was not explored. Overall, outcomes for hand function after secondary brachial plexus reconstruction are very limited from a functional perspective (20, 21). Tendon transfers were deemed not feasible due to a lack of local muscles for hand reanimation. A free gracilis transfer was discussed, however, omitted due to a lack of strong motor nerves for reinnervation, the contracted position of the hand as described above and, finally, the distinct wish of our patient against further reconstructive efforts.

For these reasons the possibility of elective amputation and subsequent prosthetic fitting was explored further with our patient. While this procedure had not been described previously in adult patients with OBPI, its benefits are reported for other patient groups. For instance, a patient suffering from arthrogryposis multiplex congenita showed an improvement of function, daily activities, independence and quality of life after prosthetic reconstruction. The prosthesis enhanced his self-confidence in terms of his appearance, which promoted enjoyment of social interactions and activities (22). Similar outcomes are reported for patients who had undergone a bionic reconstruction after severe traumatic brachial plexus injuries (9, 10). In a first case series of three patients with brachial plexus injuries where the amputation was at a transradial level the mean ARAT score (±standard deviation) increased from 5.3 ± 4.7 to 30.7 ± 14, the mean SHAP from 9.3 ± 1.5 to 65.3 ± 19.4 and the mean DASH improved from 46.5 ± 18.7 to 11.7 ± 8.4 (10). Also for five patients with more severe brachial plexus injuries who underwent an amputation above the elbow with following prosthetic fitting the mean ARAT increased from 0.6 ± 1.3 to 17.3 ± 1.5, the mean SHAP from 4 ± 3.7 to 22 ± 9.2, the mean DASH decreased from 52.5 ± 9.4 to 31.2 ± 9.8 and the mean VAS from 8.5 ± 1 to 6.7 ± 2.1 (9). Moreover, the ability to act bimanually had a positive influence on the well-being of the patients and their social interaction with others (10). Altogether, the results of these studies are comparable with our case report, indicating that bionic reconstruction can improve function and independence in daily life, as well as have a positive effect on the quality of life in selected patients.

Comparable to these other indications, the described interventions resulted in an increase of hand and arm function and enabled our patient to perform tasks of daily living, which were not possible beforehand. In contrast to patients with a brachial plexus injury in adulthood, our patient never experienced his affected hand as functional. Considering this, it is remarkable that despite the life-long lack of hand function, the patient seemed to incorporate the prosthesis very well and indicated a high level of embodiment in the questionnaire. Interestingly, the statements “I had the feeling that the prosthesis was part of my body” and “I felt the prosthesis only as a tool, and not as a part of my body” were both rated very high on the NRS. The patient explained that in his view these two statements do not exclude each other in his perception and merely depend on the situation. When he is outside and interacting with others in social situations, the prosthesis is part of his body and gives him a feeling of bodily integrity. However, when he focuses on using the prosthesis during specific functional tasks, he perceives it as a tool. Furthermore, he stated that his prosthesis is definitely not the same as the biological hand, but still belongs to him. These observations reveal the complexity of body image concepts in this specific group of patients who sacrifice parts of their (non-functional) insensate human frame for a bionic replacement. These findings indicate the importance of investigating the topic of body image and embodiment with a combination of quantitative and qualitative research methods. Comparing the quantitative scores of the prosthetic embodiment with other patients who underwent bionic reconstruction after traumatic brachial plexus injury (11), our patient had higher ratings in all items *(“I had the feeling that the prosthesis was part of my body.”, “I felt the prosthesis only as a tool, and not as a part of my body.”, “I did bimanual tasks with my intact arm/hand together with my prosthesis.”, “I felt that I had full control over the prosthesis.”, “I liked wearing the prosthesis.”, “I felt that my prosthesis looked like a real part of the body.”)*. These findings could be supported by the fact, that he now for the first time in life has a meaningful functional hand compared to the other cohort. In line with these ratings, using the prosthesis in gestures indicating a strong embodiment could be observed during the follow-up visit. They included touching the prosthesis with the unaffected hand, holding both “hands” and putting both “hands” in the trouser pocket.

The long follow-up period of 1.5 years after amputation is a strength of this case report. This period gives a good insight into the long-term outcomes of the final prosthesis use. In addition, the choice of assessments and questionnaires allows a holistic/exhaustive picture of the outcomes. The assessments were performed and scored by two experienced therapists and video recorded to increase reliability.

As this is a case report, we cannot generalize the results obtained from this one patient, however it does provide evidence that in severe cases of OBPI this concept will provide solid hand function with a high level of embodiment.

Patient selection as well as education and professional support through an experienced multidisciplinary team (including surgeons, occupational/physical therapists, psychologists and prosthetists) during the whole process are essential. A tailored psychosocial assessment and a structured rehabilitation program have proven very helpful in our experience. However, bionic reconstruction should only be performed, when biological restoration and rehabilitation measures have been exhausted and no other option is available. More research should explore the reconstructive and rehabilitative options for adults suffering OBPI, as this patient cohort is currently underrepresented in literature.

## Patient Perspective

The patient's quality of life has improved, as he is able to do things with the prosthesis he could not do before. Accordingly, he feels assured in his decision and would undergo bionic reconstruction again, maybe even at an earlier point.

## Data Availability Statement

The original contributions presented in the study are included in the article/Supplementary Material, further inquiries can be directed to the corresponding author.

## Ethics Statement

The studies involving human participants were reviewed and approved by Ethics Committee, Medical University of Vienna. The patients/participants provided their written informed consent to participate in this study. Written informed consent was obtained from the individual(s) for the publication of any potentially identifiable images or data included in this article.

## Author Contributions

CG, AS, OCA, and AB conceived and designed the study. AS, AB, AP, and CG performed the data acquisition and interpreted the data. AB, AS, CG, AP, OCA, and GL wrote and edited the manuscript. All authors gave final approval for publication.

## Funding

This project received funding from the European Research Council (ERC) under the European Union's Horizon 2020 Research and Innovation Program (Grant Agreement No. 810346).

## Conflict of Interest

The authors declare that the research was conducted in the absence of any commercial or financial relationships that could be construed as a potential conflict of interest.

## Publisher's Note

All claims expressed in this article are solely those of the authors and do not necessarily represent those of their affiliated organizations, or those of the publisher, the editors and the reviewers. Any product that may be evaluated in this article, or claim that may be made by its manufacturer, is not guaranteed or endorsed by the publisher.

## Supplementary Material

Supplementary Material 1 CARE checklist.

Supplementary Material 2 Raw data of DASH, ARAT and SHAP before the amputation, with the hybrid prosthesis and with the final fitting and raw data of the embodiment questions.

Supplementary Material 3 Videos from ARAT Exercise “Block, 5 cm^3^” with the plexus hand, the hybrid prosthesis and the final prosthesis (1.5 years follow-up).
