# Supplementary material for: Case Report: Bionic Reconstruction in an Adult With Obstetric Brachial Plexus Injury
Source: Front Rehabil Sci. 2022 Jan 5;2:804376. doi: 10.3389/fresc.2021.804376 (PMC9397750; doi:10.3389/fresc.2021.804376)
Supplement: Supplementary Material 1 — CARE checklist. [file Data_Sheet_1.PDF]

CARE case report guidelines

CARE Checklist of information to include when writing a case report

BY-NC-ND

Topic	Item	Checklist item description	Reported on Line
Title	1	The diagnosis or intervention of primary focus followed by the words "case report"	in Title
Key Words	2	2 to 5 key words that identify diagnoses or interventions in this case report, including "case report"	13-14*
Abstract (no references)	3a	Introduction: What is unique about this case and what does it add to the scientific literature?	16-23
	3b	Main symptoms and/or important clinical findings	24-35
	3c	The main diagnoses, therapeutic interventions, and outcomes	24-39
	3d	Conclusion—What is the main "take-away" lesson(s) from this case?	37-41
Introduction	4	One or two paragraphs summarizing why this case is unique (**may include references**)	49-65
Patient Information	5a	De-identified patient specific information.	67-70
	5b	Primary concerns and symptoms of the patient.	72-73
	5c	Medical, family, and psycho-social history including relevant genetic information	67-72
	5d	Relevant past interventions with outcomes	68-73
Clinical Findings	6	Describe significant physical examination (PE) and important clinical findings.	75-78; 83-90
Timeline	7	Historical and current information from this episode of care organized as a timeline	Figure 1
Diagnostic Assessment	8a	Diagnostic testing (such as PE, laboratory testing, imaging, surveys).	76-78; 83-90
	8b	Diagnostic challenges (such as access to testing, financial, or cultural)	N/A
	8c	Diagnosis (including other diagnoses considered)	67
	8d	Prognosis (such as staging in oncology) where applicable	101-104; 109-110; 113-115, 131-132
Therapeutic Intervention	9a	Types of therapeutic intervention (such as pharmacologic, surgical, preventive, self-care)	91-149
	9b	Administration of therapeutic intervention (such as dosage, strength, duration)	79-80; 148-149
	9c	Changes in therapeutic intervention (with rationale)	N/A
Follow-up and Outcomes	10a	Clinician and patient-assessed outcomes (if available)	150-174
	10b	Important follow-up diagnostic and other test results	150-174
	10c	Intervention adherence and tolerability (How was this assessed?)	148-149
	10d	Adverse and unanticipated events	174
Discussion	11a	A scientific discussion of the strengths AND limitations associated with this case report	232-238
	11b	Discussion of the relevant medical literature **with references**.	176-231
	11c	The scientific rationale for any conclusions (including assessment of possible causes)	176-231
	11d	The primary "take-away" lessons of this case report (without references) in a one paragraph conclusion	239-246
Patient Perspective	12	The patient should share their perspective in one to two paragraphs on the treatment(s) they received	247-250
Informed Consent	13	Did the patient give informed consent? Please provide if requested	Yes ☒ No ☐

* according to the journal 5 to 8 keywords are allowed
