# Supplementary material for: Case Report: Bionic Reconstruction in an Adult With Obstetric Brachial Plexus Injury
Source: Front Rehabil Sci. 2022 Jan 5;2:804376. doi: 10.3389/fresc.2021.804376 (PMC9397750; doi:10.3389/fresc.2021.804376)
Supplement: Supplementary Material 2 — Raw data of DASH, ARAT and SHAP before the amputation, with the hybrid prosthesis and with the final fitting and raw data of the embodiment questions. [file Table_1.pdf]

Supplementary Material - Raw data of DASH, ARAT and SHAP before the amputation, with the hybrid prosthesis and with the final fitting and raw data of the embodiment questions

1. **Raw Data of the plexus hand, the hybrid prosthesis and the 1.5 years follow-up with the prosthesis of the Action Research Arm Test (ARAT)**

Tasks ARAT	Plexus hand	Hybrid prosthesis	1.5 years follow-up with prosthesis
Grasp subscale			
Block, 10 cm^3	0	0	0
Block, 2.5 cm^3	2	2	2
Block, 5 cm^3	2	2	2
Block, 7.5 cm^3	0	2	2
Cricket ball	0	1	2
Sharpening stone	0	2	2
Grip subscale			
Pour water from one glass to another	1	1	1
Displace 2.25-cm alloy tube from one side of the table to the other	1	1	2
Displace 1-cm alloy tube from one side of the table to the other	2	1	2
Push washer over bolt	0	0	0
Pinch subscale			
Ball bearing, held between ring finger and thumb	0	0	0
Marble, held between index finger and thumb	1	2	2
Ball bearing, held between middle finger and thumb	0	0	0
Ball bearing, held between index finger and thumb	0	0	0
Marble, held between ring finger and thumb	0	0	0
Marble, held between middle finger and thumb	0	0	0
Gross movement subscale			
Hand to behind the neck	1	1	1

Hand to top of head	1	1	1
Hand to mouth	1	1	1
**Total (maximal score = 57)**	**12**	**17**	**20**

Scoring: 3 = normal performance; 2 = task completed, but with great difficulty or abnormally slowly; 1 = task partially completed; 0 = not able to complete any part of the task within 60 seconds

2. Raw Data of the plexus hand, the hybrid prosthesis and the 1.5 years follow-up with the prosthesis of the Southampton Hand Assessment Procedure (SHAP)

Tasks SHAP	Plexus hand	Hybrid prosthesis	1.5 years follow-up with prosthesis
Abstract Objects			
Light Sphere	15.75	14.07	6.06
Light Tripod	22.78	22.23	8.82
Light Power	21.75	6.97	8.50
Light Lateral	17.88	9.53	9.56
Light Tip	36.03	12.34	10.72
Light Extension	98.00	25.44	7.60
Heavy Sphere	100.00	13.17	9.56
Heavy Tripod	57.57	21.38	7.03
Heavy Power	100.00	10.84	8.38
Heavy Lateral	100.00	10.25	9.08
Heavy Tip	100.00	11.26	9.37
Heavy Extension	100.00	9.16	9.34
Activities of Daily Living (ADLs)			
Pick Up Coins	100.00	100.00	100.00
Button Board	100.00	100.00	100.00
Simulated Food Cutting	100.00	100.00	100.00

Page Turning	18.72	22.17	14.31
Jar Lid	70.66	100.00	15.56
Glass Jug Pouring	100.00	100.00	21.28
Carton Pouring	100.00	100.00	100.00
Lifting a Heavy Object	28.34	14.41	11.57
Lifting a Light Object	100.00	10.28	10.47
Lifting a Tray	7.15	8.60	9.44
Rotate Key	100.00	12.67	13.22
Open/Close Zip	100.00	100.00	11.97
Rotate A Screw	100.00	100.00	100.00
Door Handle	14.03	6.81	6.47
**Index of Function Score (maximal score = 100)**	**8**	**19**	**29**

The time for completing each the task was measured (in seconds). If a task could not be completed or lasted longer than 100.00 seconds, a time of 100.00 was documented.
The index of function was calculated on the website (http://www.shap.ecs.soton.ac.uk/entry.php).

3. Raw Data pre amputation and 1.5 years follow-up of the Disabilities of the Arm, Shoulder and Hand Questionnaire (DASH)

Questions DASH	Pre amputation	1.5 years follow-up
Open a tight or new jar.	3	1
Write.	1	1
Turn a key.	1	1
Prepare a meal.	2	1
Push open a heavy door.	4	1
Place an object on a shelf above your head.	1	1
Do heavy household chores (e.g., wash walls, wash floors).	4	1
Garden or do yard work.	3	1
Make a bed.	2	1
Carry a shopping bag or briefcase.	2	1

Carry a heavy object (over 10 lbs).	4	2
Change a lightbulb overhead.	4	1
Wash or blow dry your hair.	2	1
Wash your back.	4	2
Put on a pullover sweater.	3	1
Use a knife to cut food.	4	2
Recreational activities which require little effort (e.g., cardplaying, knitting, etc.).	4	1
Recreational activities in which you take some force or impact through your arm, shoulder or hand (e.g., golf, hammering, tennis, etc.).	3	1
Recreational activities in which you move your arm freely (e.g., playing frisbee, badminton, etc.).	3	1
Manage transportation needs (getting from one place to another).	3	1
Sexual activities.	3	1
During the past week, to what extent has your arm, shoulder or hand problem interfered with your normal social activities with family, friends, neighbours or groups?	4	2
During the past week, were you limited in your work or other regular daily activities as a result of your arm, shoulder or hand problem?	4	2
Please rate the arm, shoulder or hand pain.	3	1
Please rate the arm, shoulder or hand pain when you performed any specific activity.	3	1
Please rate the tingling (pins and needles) in your arm, shoulder or hand.	4	2
Please rate the weakness in your arm, shoulder or hand.	4	3
Please rate the stiffness in your arm, shoulder or hand.	3	1
During the past week, how much difficulty have you had sleeping because of the pain in your arm, shoulder or hand?	1	4

I feel less capable, less confident or less useful because of my arm, shoulder or hand problem.	4	4
**Total (no disability = 0; maximal disability = 100)**	**50**	**11.7**

Scoring: 1 = no difficulty/not at all; 2 = mild difficulty/slightly; 3 = moderate difficulty/moderately; 4 = severe difficulty/quite a bit; 5 = unable/extremely

The total score as reported in the main manuscript is calculated in the following way: sum of all responses, divided trough 30, subtract 1, and multiplate this score with 25.

4. Raw Data 1.5 years follow-up questions regarding embodiment

Questions	NRS
"I had the feeling that the prosthesis was part of my body."	10
"I felt the prosthesis only as a tool, and not as a part of my body."	9
"I did bimanual tasks with my intact arm/hand together with my prosthesis."	9
"I felt that I had full control over the prosthesis."	9
"I liked wearing the prosthesis."	9
"I felt that my prosthesis looked like a real part of the body."	9

Scoring: 0 = never; 10 = always; NRS = numeric rating scale
